# Immunomodulatory mediators IL-33, soluble ST2, IL-10, IFN-*γ* in the serum of patients with oral potentially malignant disorders and oral squamous cell carcinoma

**DOI:** 10.3389/froh.2026.1793916

**Published:** 2026-04-20

**Authors:** Swetha Acharya, Usha Hegde, Subba Rao Venkata Madhunapantula, Medha Karnik, Anirudh Balakrishna Acharya, Pushparaja Shetty

**Affiliations:** 1 Department of Oral Pathology and Microbiology, JSS Dental College and Hospital, JSS Academy of Higher Education and Research (JSSAHER), Mysuru, India; 2 Center of Excellence in Molecular Biology and Regenerative Medicine (CEMR) Laboratory (DST–FIST supported center and ICMR Collaborating Center of Excellence– ICMR–CCoE), Department of Biochemistry (DST–FIST supported department), JSS Medical College & Hospital, JSSAHER, Mysuru, India; 3 Department of Restorative Dentistry, College of Dental Medicine, University of Sharjah, Sharjah, United Arab Emirates; 4 Department of Oral & Maxillofacial Pathology & Oral Microbiology, A.B. Shetty Memorial Institute of Dental Sciences, NITTE (Deemed to be University), Mangaluru, India

**Keywords:** interferon gamma (IFN-γ), interleukin-10 (IL-10), interleukin-33 (IL-33), interleukin-33 receptor (IL-33R), oral squamous cell carcinoma (OSCC), suppression of tumorigenicity 2 (ST2), soluble suppression of tumorigenicity 2 (sST2)

## Abstract

**Background:**

Interleukin-33 (IL-33) and its receptor Suppression of tumorigenicity 2 (ST2), along with IL-10 and IFN-*γ*, exert contrasting roles in tumor growth, immune evasion, and host defense. IL-33/ST2 signalling can either promote tumor progression or, in some contexts, enhance anti-tumor immunity. However, the role of serum IL-33 and soluble ST2 in Oral squamous cell carcinoma (OSCC) or its precursor, Oral potentially malignant disorders (OPMDs), remains largely unexplored.

**Aims:**

To evaluate whether IL-33 and ST2, in conjunction with their interactions with IL-10 and IFN-*γ*, influence tumor dynamics in OSCC, as reflected in their serum levels.

**Materials and methods:**

Ninety participants were enrolled in this cross-sectional observational study and divided into three groups: Healthy controls (HC) (*n* = 30), OPMDs (*n* = 30), and OSCC (*n* = 30). Clinicopathological data were recorded, and 5 mL of venous blood was collected from each subject prior to treatment. Serum IL-33, soluble ST2, IL-10, and IFN-*γ* levels were quantified using ELISA. The data were analysed by applying the Kruskal–Wallis/Mann–Whitney *U*-tests, Receiver operating characteristics (ROC) curve analysis, DeLong's test and binomial logistic regression (BLR), with significance set at *p* < 0.05.

**Results:**

The analysis demonstrated a progressive and significant increase in IL-33, ST2, and IL-10 levels from HC to OPMDs to OSCC (*p* *<* *0.001*). In contrast, IFN-*γ* levels exhibited a significant inverse trend, being highest in OPMDs, comparable in HC, and lowest in OSCC (*p* *<* *0.001*). Immune mediators in OSCC showed significant associations with clinicopathological parameters, including tumor stage, depth of invasion, lymph nodal metastasis (LNM), tumor budding, and surgical margin status (*p* < 0.05). Serum IL-10 was the strongest positive predictor, effectively discriminating lymph node status (LNS). IL-33 and soluble ST2 showed positive trends toward predicting LNM with high classification accuracy. ROC analysis showed excellent discriminatory ability of all immunomodulatory mediators for distinguishing OSCC from OPMDs. Although soluble ST2 had the highest AUC, DeLong's test (*p* = 0.592) showed no significant difference, indicating comparable diagnostic potential. BLR confirmed their diagnostic relevance, with elevated IL-33, soluble ST2, and IL-10 increasing the odds of OSCC, while higher IFN-*γ* reduced the risk.

**Conclusion:**

The panel of immunomodulatory mediators analysed here reflects a biologically relevant shift toward pro-tumorigenic inflammation and immune evasion, underscoring their role as biomarkers of malignant progression. Collectively, these immunomodulatory mediators demonstrated strong diagnostic accuracy in differentiating OSCC from OPMDs and showed potential for risk stratification.

## 1 Introduction

Oral potentially malignant disorders (OPMDs) represent a heterogeneous group of mucosal abnormalities with an increased risk of malignant transformation (MT) into oral squamous cell carcinoma (OSCC), which constitutes nearly 95% of all oral cavity cancers and is a major contributor to the global burden of head and neck squamous cell carcinoma (HNSCC) (1, 2). The overall MT rate among all types of OPMDs is 7.9% (3). Despite advances in clinical and histopathological assessment, predicting which OPMDs will undergo MT remains challenging due to the lack of standardized and reliable risk—stratification methods (4). Given the high morbidity, mortality, and poor five-year survival associated with OSCC, early detection and risk assessment are crucial (2). In this context, the evaluation of biomarkers-particularly immune-related biomarkers such as cytokines—has gained increasing importance. Cytokines play a central role in inflammation, immune dysregulation, tumor initiation, and progression, and alterations in their expression may reflect early molecular changes preceding overt malignancy. Assessing cytokine profiles in systemic fluids such as blood, plasma, or serum may provide a more stable and representative measure of host immunosurveillance than saliva, which can be influenced by local oral inflammatory conditions (5, 6). Therefore, identifying and validating cytokine-based biomarkers in OPMDs and OSCC holds significant promise for improving early diagnosis, predicting MT and prognostication. Although serum cytokines are generally considered more stable and less susceptible to immediate environmental variations than saliva, they may still be influenced by systemic inflammatory conditions, infections, comorbidities, and other local inflammatory processes. However, to a considerable extent, these confounding effects can be minimized through stringent selection criteria.

The occurrence of cancer is often accompanied by immune evasion and tumor-promoting inflammation, with interleukins (ILs) playing a pivotal role in the immune-inflammatory mechanism (7). Various ILs actively participate in intricate tumor regulatory networks, impacting tumor progression via diverse mechanisms. Clinical investigations have identified abnormal serum cytokine levels in cancer patients, with these markers strongly linked to tumor invasion, metastasis, treatment response, postoperative recurrence, and prognosis. Nevertheless, the precise role of several serum ILs in cancer biology remains incompletely understood (7). Among them, Interleukin-33 (IL-33) has gained increasing attention as a key mediator of tumorigenesis and tumor–immune crosstalk, with accumulating evidence implicating its involvement in cancer progression (8, 9).

IL-33, a member of the IL-1 cytokine family identified in 2005, exerts its biological effects through the Suppression of tumorgenicity 2 (ST2) receptor, which exists as both a transmembrane signaling form (ST2L) and a soluble decoy receptor (sST2) (10–12). IL-33 is a nuclear cytokine expressed by a wide range of cells, including endothelial cells, fibroblasts, epithelial cells, and immune cells such as dendritic cells and macrophages (10, 11). It is released in response to cellular stress, necrosis, mechanical or oxidative damage, or cell activation, thereby functioning as an alarmin. Upon tissue injury or necrosis, IL-33 binds to the ST2 receptor complex and activates downstream signaling pathways. These signaling cascades promote diverse biological effects (13–16). Via activation of ST2L, IL-33 can induce either helper T1 (Th1) Immune responses (IR)—characterized by increased production of IFN-*γ*, TNF-α, IL-2, and IL-12 or helper T2(Th2) IR, accompanied by the secretion of IL-4, IL-5, IL-6, IL-10, and IL-13. Notably, IL-33 is considered a potent driver of Th2 immunity (6, 17, 18), and its role in cancer is highly context-dependent, exhibiting both pro-tumorigenic and anti-tumor activities (19, 20). In contrast, soluble ST2 attenuates IL-33 signaling by acting as a decoy receptor that limits IL-33 bioavailability and biological activity (12). Collectively, IL-33 and ST2 represent key regulators of IR and have emerged as critical contributors to cancer progression and immune modulation (16, 20).

IL-33 and IL-10 are functionally interconnected within the inflammatory network, as IL-33 can induce downstream IL-10 production. The IL-33/ST2 axis has been shown to drive the polarization of naïve T cells toward a Th2 phenotype, while blockade of this pathway enhances anti-tumor Th1 IR (21). In HNSCC, disease progression is linked to reduced Th1 cytokines alongside elevated Th2 cytokines, facilitating immune evasion and tumor growth. Among Th2 cytokines, IL-10 plays a central immunosuppressive role (22, 23).

Emerging evidence indicates that IL-33 can also promote type 1 IR and exert anti-tumor effects (21, 24). Activation of the IL-33/ST2 pathway can enhance Th1 IR, characterized by increased production of Th1 cytokines. IFN-*γ* suppresses type 2 IR and supports Th1 dominance (21, 25). In addition, IL-33/ST2 signaling promotes the expansion and activation of natural killer (NK) cells, leading to IFN-*γ*–mediated tumor cell killing (21). Collectively, the balance between Th1-associated cytokines (e.g., IFN-*γ*) and Th2-associated cytokines (e.g., IL-10) is a critical determinant of tumor progression or regression (26, 27). Thus, IL-33 and its receptors, including membrane-bound ST2 and soluble ST2 (sST2), regulate multiple dimensions of cancer immunity and tumor biology. IL-33 as a cytokine with dual functionality, IL-33 engages both immunosuppressive (IL-10–linked) and pro-inflammatory (IFN-*γ*–associated) circuits, positioning it as an important mediator within the TME.

Beyond the TME, IL-33 is detectable in the circulation (14), and elevated serum and tissue levels have been reported in several malignancies, including gastric, lung, hepatocellular, and colorectal cancers, where they correlate with adverse prognostic factors (8, 9, 28–32). In contrast, data regarding soluble ST2 in cancer patients remain limited and inconsistent (12), although increased levels of these mediators have been linked to prognostic indicators across multiple tumor types. A recent review integrating available evidence identified serum IL-33 and soluble ST2 as emerging diagnostic and risk-stratification biomarkers in cancer, underscoring the growing relevance of the IL-33/ST2 axis in cancer biology and clinical assessment. Notably, this review revealed a substantial lack of studies specifically examining circulating IL-33 and soluble ST2 in OSCC, representing a significant gap in the current literature (33).

A recent systematic review examined the diagnostic and prognostic significance of differential IL-33 and ST2 expression in tumor tissues as emerging biomarkers in HNSCC, concluding that IL-33 may represent a promising prognostic biomarker and potential therapeutic target. The results further suggested that IL-33 and ST2 expression in HNSCC tumor tissues predominantly promotes tumor progression rather than exerting inhibitory effects. However, the review highlighted that the clinical significance of circulating IL-33 and ST2 in HNSCC remains insufficiently explored and requires further investigation (34).

Hence, the aim of the present study is to measure and compare serum levels of IL-33 and ST2 among healthy individuals, patients with OPMDs, and those with OSCC. Analysis of these data is expected to elucidate the relationship between IL-33 and ST2 in OPMDs and OSCC, and to assess their associations with clinicopathological characteristics (CPC) of OSCC, thereby evaluating their potential as cancer biomarkers. Concurrent measurement of serum IL-10 and IFN-*γ* will further clarify their behaviour and interactions with IL-33 and ST2 in OPMDs and OSCC. Overall, this study investigates the hypothesis that whether IL-33 and ST2, in conjunction with IL-10 and IFN-*γ*, influence disease dynamics, including progression or regression, as reflected in their serum levels.

## 2 Materials and methods

The study was conducted at a tertiary care teaching hospital. The research protocol was approved by the Institutional Ethics Committee (IEC) of the participating institution (JSSDCH/IEC/Research Protocol No: 22/2023, dated 20.05.2023). This cross-sectional observational study was carried out from May 2023 through July 2025. Recruitment of study participants was undertaken at appropriate locations within the selected institutions/facilities. Study participants were enrolled by professionally qualified and experienced clinicians. The volunteers recruited for this study included healthy individuals and patients visiting the institution and its associated hospitals. Written informed consent was obtained from all participants in accordance with ethical guidelines, and the study was conducted in compliance with the Declaration of Helsinki. Participant anonymity was maintained throughout the research process.

### 2.1 Source of data

The study included ninety participants recruited between 2023 and 2025 through convenience sampling, comprising thirty healthy controls (HC), thirty patients with OPMDs, and thirty patients with OSCC. Data collection included demographic, clinical, and histopathological details (where applicable), along with serum samples for the estimation of selected immunomodulatory mediators. The control group consisted of thirty age-matched healthy individuals without systemic illness or oral lesions, with serum samples obtained after confirming the absence of oral complaints. The OPMDs group comprised thirty patients diagnosed with leukoplakia (LKP; *n* = 15) or oral submucous fibrosis (OSF; *n* = 15), with or without dysplasia. Pre-treatment clinicopathological details were documented, and serum samples were collected. The OSCC group included thirty biopsy-confirmed patients, from whom clinicopathological data were recorded and pre-treatment serum samples were obtained.

In patients with the OPMDs, following demographic and clinicopathological data were recorded: age, gender, habits, site, extent of lesion, type, degree of dysplasia. In OSCC patients, the following demographic and clinicopathological data were recorded: age, gender, habits, tumor site, tumor extent, association with OPMDs, lesion type, tumor size (TS), tumor staging (TNM), (AJCC, 8th Edition), tumor grading, (Broders’ Grading), tumor depth(TD) (35), lymph node metastasis (LNM), tumor budding (TB) (36), margin status, tumor volume (TV) (37), [The tumor volume—manually measuring tumor length (L), width (W), and height (H)—and then calculated the TV using the formula 0.5236 × L × W × H-based on Imaging reports], local and regional recurrence and patient survival details were additionally recorded through follow-up.

### 2.2 Selection criteria

Participants were selected based on defined inclusion and exclusion criteria: The healthy controls (HC) group (Group 1) included systemically healthy volunteers above 18 years of age, without current or past adverse habits such as tobacco or arecanut use, not on medications such as immunosuppressants, steroids, NSAIDs, or antidepressants, and without periodontal disease, oral infections, OPMDs, or OSCC. Participants undergoing routine dental examinations, restorative procedures, minor oral surgical interventions, or extractions for orthodontic purposes or impacted teeth were considered eligible. Exclusion criteria included pregnant, lactating, or menopausal women and those unwilling to provide informed consent. The OPMDs (Group 2) included patients diagnosed with LKP or OSF, with or without dysplasia, who had no systemic illness, comorbid conditions, or prescribed medications (immunomodulators, steroids, NSAIDs, or antidepressants); unwilling participants were excluded. The OSCC (Group 3) included histopathologically confirmed cases of OSCC without comorbid conditions or prescribed medications (immunomodulators, steroids, NSAIDs, or antidepressants). Exclusion criteria included OSCC patients with chronic illness, allergic conditions, acute infections, systemic diseases, chronic lung disease, rheumatic or autoimmune diseases, prior chemotherapy or radiotherapy, previous extra-oral malignancies, recurrent OSCC, histological variants such as verrucous, basaloid, or spindle cell carcinoma, and refusal to provide informed consent.

### 2.3 Sample size estimation

As no prior studies have evaluated serum IL-33/ST2 in OPMDs and OSCC, sample size estimation was based on the gastric cancer study by Sun et al. (8). Using a two-tailed *t*-test (effect size d = 0.8477, *α* = 0.05, powe*r* = 0.8, allocation ratio = 1.19), the required sample size was calculated as 78 participants (26 per group) with an actual power of 0.8172 using G*Power software.

### 2.4 Sampling technique

A total of ninety study samples were selected using convenience sampling.

### 2.5 Sample collection and preparation

Peripheral venous blood samples (5 mL) were collected from participants, including HC, prior to initiation of treatment or surgery, from the antecubital vein using sterile 21-gauge BD Vacutainer® needles and serum separator tubes (SST; BD Vacutainer®, BD Biosciences, Oxford, UK) under sterile conditions. Following collection, the blood samples were allowed to clot undisturbed at room temperature for 30 min. Serum samples were then segregated by centrifugation at 1,000×g for 10 min, carefully aliquoted into sterile polypropylene microtubes, and immediately stored at −80 °C until analysis. This procedure was uniformly followed for all participants, including HC. Frequent freeze–thaw cycles were stringently avoided to preserve cytokine integrity, stability and reliability of downstream assays.

### 2.6 Cytokine measurement by ELISA

Serum levels of IL-33, ST2/IL-33R, IL-10, and IFN-*γ* were quantified using commercially available enzyme-linked immunosorbent assay (ELISA) kits (DuoSet® ELISA; R&D Systems, Minneapolis, MN, USA, Cat.No: DY3625B-05, DY523B-05, DY217B-05, DY285B-05). All assays were performed in duplicate in strict accordance with the manufacturers’ instructions. Results were expressed in picograms per milliliter (pg/mL). The ELISA was conducted using a sandwich technique based on monoclonal antibody binding. Undiluted serum samples were allowed to react with monoclonal antibodies specific for epitopes of human IL-33, ST2/IL-33R, IL-10, and IFN-*γ*. Upon completion of the assay protocol, absorbance values were measured at 450 nm using a multimode microplate reader (Enspire® 2300, PerkinElmer Inc., Waltham, MA, USA). Cytokine concentrations were quantified by comparing the absorbance of each sample with a standard curve. The standard curve was generated by plotting known concentrations of cytokine standards on the *x*-axis against their corresponding absorbance values on the *y*-axis. Cytokine concentrations were calculated by interpolating sample absorbance values from the standard calibration curve generated using Microsoft Excel. Linearity was assessed by the coefficient of determination (*R*^2^). Concentrations of unknown samples were calculated using the linear regression equation derived from the standard curve (y = mx + c). The R² values ranged from 0.97 to 0.99 (IL-33: 0.999, soluble ST2: 0.973, IL-10: 0.976, IFN-*γ*: 0.967), indicating good linearity of the standard curves within the working range.

### 2.7 Statistical analysis

Statistical analyses were conducted using IBM SPSS Statistics for Windows, version 20.0 (IBM Corp., Armonk, NY, USA) and jamovi version 2.6.26 (The jamovi project, Sydney, Australia); normality was assessed with the Shapiro–Wilk test, group differences with Kruskal–Wallis/Mann–Whitney *U*-tests, and correlations among immunomodulatory mediators in each group were assessed with Spearman's correlation. Receiver operating characteristics (ROC) curve analysis to determine optimal cut-off values and the Area Under the Curve (AUC) with 95% confidence intervals, the DeLong's test for AUC comparison, and binomial logistic regression (BLR) analysis were conducted to identify independent predictors using jamovi. Statistical significance was set at *p* < 0.05.

## 3 Results

Serum IL-33 was measurable in 67% of HC and 90% of patients with OPMDs. In contrast, IL-33 was measurable in all OSCC patients, although concentrations were generally close to the lower limit of the assay's analytical range. Serum ST2 and IFN-*γ* were measurable in all participants. The HC exhibited the lowest serum IL-10 levels, with measurable concentrations observed in only 43% of individuals. In comparison, IL-10 was measurable in all OPMDs and OSCC patients; however, levels in these groups were largely clustered near the lower limit of the assay's analytical range.

**Figure 1:**
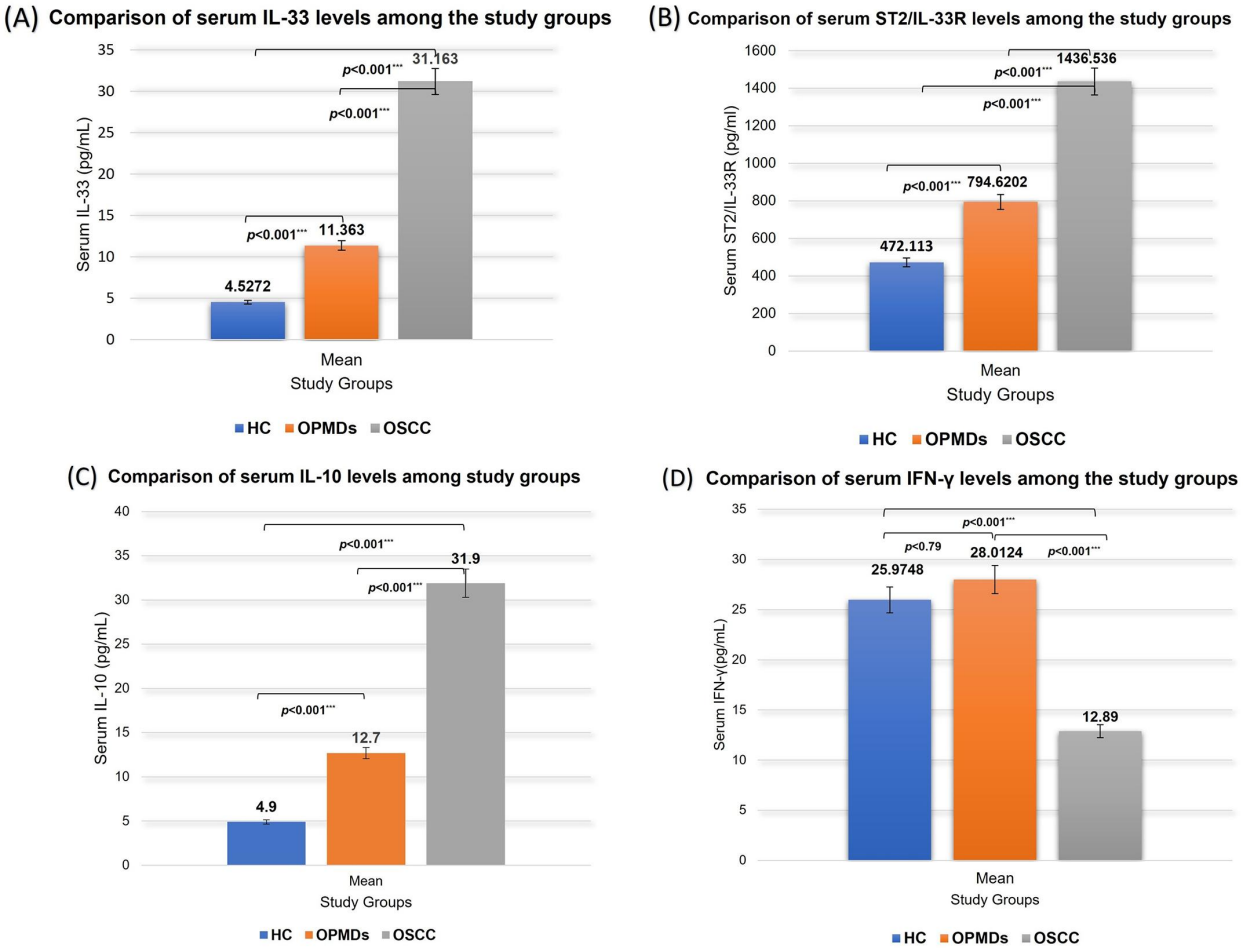
Comparison of serum levels of **(A)** IL-33, **(B)** soluble ST2, **(C)** IL-10, and **(D)** IFN-*γ* among healthy controls (HC), OPMDs, and OSCC cohorts using the Kruskal–Wallis test, followed by pairwise Mann–Whitney *U*-tests for group comparisons.

Comparative analysis of serum cytokine levels demonstrated a consistent and statistically significant stepwise increase in IL-33, soluble ST2, and IL-10 concentrations from HC to OPMDs and OSCC patients (Figure 1). Mean serum IL-33 levels increased from 4.53 ± 3.94 pg/mL in HC to 11.4 ± 7.08 pg/mL in OPMDs and 31.2 ± 8.94 pg/mL in OSCC, while serum ST2 levels rose from 472 ± 147 pg/mL to 795 ± 233 pg/mL and 1,437 ± 225 pg/mL, respectively. Similarly, serum IL-10 concentrations were lowest in HC (4.92 ± 1.03 pg/mL), intermediate in OPMDs (12.7 ± 4.78 pg/mL), and highest in OSCC patients (31.9 ± 13.2 pg/mL). Kruskal–Wallis analysis revealed highly significant differences among the three groups for all immune mediators (*p* < 0.001), and pairwise comparisons confirmed significant differences between each group (HC vs. OPMDs, OPMDs vs. OSCC, and HC vs. OSCC; all *p* < 0.001). Collectively, these findings demonstrate a progressive elevation of IL-33, soluble ST2, and IL-10 with advancing disease, supporting their potential utility as indicators of MT and tumor progression.

Serum IFN-*γ* concentrations between the study groups showed significant variation (*p* < 0.001). The mean concentration was highest in OPMDs patients (28.0 ± 11.2 pg/mL), followed by HC (26.0 ± 6.22 pg/mL), and was markedly reduced in OSCC patients (12.9 ± 2.79 pg/mL). Pairwise comparisons showed no significant difference between HC and OPMDs (*p* = 0.79), whereas both OPMDs vs. OSCC (*p* < 0.001) and OSCC vs. HC (*p* < 0.001) showed highly significant reductions in IFN-*γ* levels in OSCC. The above findings suggest that while IFN-*γ* levels remain relatively maintained in OPMDs compared to HC, its marked decline in OSCC group may indicate a role in immune suppression during cancer development.

In OSCC patients, serum IL-33, ST2, and IL-10 levels showed significant associations with multiple CPC indicative of tumor aggressiveness. Elevated IL-33 levels were significantly associated with larger TS (≥4 cm), increased TV and TD, advanced clinical and pathological TNM stages, LNM, TB, positive surgical margins, and poorer survival outcomes (all *p* < 0.05) (Table 1). Similarly, higher serum ST2 levels correlated with increased TS, TV, and TD, advanced clinical and pathological TNM stages, poorer differentiation, LNM, TB, recurrence, positive margins, and reduced survival (all *p* < 0.05) (Table 2). Serum IL-10 levels were also significantly elevated in association with advanced clinical and pathological TNM stages, greater TD, LNM, TB, and positive surgical margins (all *p* < 0.05) (Supplementary Table S1). Collectively, these findings indicate that increased circulating levels of IL-33, ST2, and IL-10 are closely associated with greater tumor invasiveness and more advanced disease characteristics in OSCC.

**Table 1:** The association between serum IL-33 levels and the CPC of OSCC.

Parameters	Category	*n* %	Serum IL-33 Mean ± SD	U-Statistic	*p-*value
Age	≤45	4 (13)	28.75 ± 8.95	41	0.501^a^
	>45	26 (87)	31.58 ± 9.03		
Sex	Male	22 (73)	30.8 ± 8.89	82	0.778^a^
	Female	8 (27)	32.13 ± 9.53		
Habits	Absent	7 (23)	31.43 ± 9.41	78.5	0.922^a^
	Present	23 (77)	31.13 ± 8.98		
Site of the lesion	Buccal Mucosa	8 (27)	27.25 ± 6.77	5.369^KWS^	0.068^b^
	Tongue	12 (40)	30.08 ± 8.94		
	Gingiva & others	10 (33)	35.7 ± 9.20		
Extension of the lesion	Single	21 (70)	29.52 ± 9.00	57.5	0.093^a^
	Multiple	9 (30)	35.11 ± 7.80		
Nature of the lesion	Exophytic	14 (47)	32.57 ± 9.88	100	0.617^a^
	Endophytic	16 (53)	30.0 ± 8.11		
Associated with OPMDs	Absent	22 (73)	31.55 ± 8.91	81	0.742^a^
	Present	8 (27)	30.25 ± 9.48		
Tumor Size	<4 cms	22 (73)	28.73 ± 8.08	38	0.019^a^^,^^c^
	≥4 cms	8 (27)	38.0 ± 7.83		
cTNM	Early (I&II)	11 (37)	26.27 ± 5.71	53	0.026^a^^,^^c^
	Advanced (III & IV)	19 (63)	34.05 ± 9.30		
Tumor Volume	≤5 cm^3^	17 (57)	26.88 ± 6.92	43.5	0.005^a^^,^^c^
	>5 cm^3^	13 (43)	36.85 ± 8.20		
Depth	≤1 cm	17 (57)	27.71 ± 6.89	60.5	0.036^a^^,^^c^
	>1 cm	13 (43)	35.77 ± 9.43		
Grade	Well-Moderately differentiated	18 (60)	28.78 ± 8.83	58	0.034^a^^,^^c^
	Moderate-Poorly differentiated	12 (40)	34.83 ± 8.06		
pLNS	pN−	18 (60)	25.33 ± 5.23	6	<0.001^a^^,^^c^
	pN+	12 (40)	40.0 ± 5.15		
pTNM	Early	15 (50)	24.93 ± 5.36	17.5	<0.001^a^^,^^c^
	Advanced	15 (50)	37.47 ± 7.2		
TB	Absent	15 (50)	27.8 ± 7.37	65	0.048^a^^,^^c^
	Present	15 (50)	34.6 ± 9.25		
PNI	Absent	19 (63)	31.47 ± 8.92	98.5	0.796^a^
	Present	11 (37)	30.73 ± 9.32		
Recurrence	Absent	20 (67)	29.1 ± 7.95	58.5	0.067^a^
	Present	10 (33)	35.4 ± 9.66		
Surgical Margins	Negative	25 (83)	29.52 ± 8.70	18	0.013^a^^,^^c^
	Positive	5 (17)	39.6 ± 4.03		
Survival	Dead	9(30)	35.78 ± 6.01	50	0.043^a^^,^^c^
	Alive	21(70)	29.24 ± 9.34		

a Mann-Whitney U.

b One-way Anova-Kruskal-Wallis test.

c Significance.

KWS, Kruskal–Wallis test statistics; cTNM, Clinical staging; pLNS, Pathological Lymph node status; pTNM, Pathological staging; PNI, Perineural Invasion; TB, Tumor budding.

**Table 2:** The association between serum ST2 levels and the CPC of OSCC.

Parameters	Category	*n* %	Serum ST2 Mean ± SD	U-statistic	*p-*value
Age	≤45	4 (13)	1547.25 ± 132.85	34.5	0.286^a^
	>45	26 (87)	1419.50 ± 233.21		
Sex	Male	22 (73)	1421.91 ± 219.94	75	0.542^a^
	Female	8 (27)	1476.75 ± 249.46		
Habits	Absent	7 (23)	1408.00 ± 278.31	78.5	0.713^a^
	Present	23 (77)	1445.22 ± 212.88		
Site of the lesion	Buccal Mucosa	8 (27)	1337.88 ± 214.79	4.594^KWS^	0.101^b^
	Tongue	12 (40)	1404.25 ± 159.76		
	Gingiva & others	10 (33)	1554.20 ± 265.96		
Extension of the lesion	Single	21 (70)	1397.71 ± 220.77	60.5	0.124^a^
	Multiple	9 (30)	1527.11 ± 220.47		
Nature of the lesion	Exophytic	14 (47)	1468.50 ± 263.10	100	0.618^a^
	Endophytic	16 (53)	1408.56 ± 190.17		
Associated with OPMDs	Absent	22 (73)	1439.23 ± 215.06	85	0.888^a^
	Present	8 (27)	1429.13 ± 266.54		
Tumor Size	≤4 cms	22 (73)	1373.41 ± 204.32	32.5	0.009^a^^,^^c^
	>4 cms	8 (27)	1610.13 ± 194.11		
cTNM	Early (I&II)	11 (37)	1286.36 ± 135.59	39	0.005^a^^,^^c^
	Advanced (III & IV)	19 (63)	1523.47 ± 223.82		
Tumor Volume	≤5 cm^3^	17 (57)	1332.24 ± 186.84	37.5	0.002^a^^,^^c^
	>5 cm^3^	13 (43)	1572.92 ± 200.80		
Depth	≤1 cm	17 (57)	1340.53 ± 181.54	44.5	0.006^a^^,^^c^
	>1 cm	13 (43)	1562.08 ± 219.90		
Grade	Well-Moderately differentiated	18 (60)	1364.61 ± 188.26	58	0.034^a^^,^^c^
	Moderate-Poorly differentiated	12 (40)	1544.42 ± 240.05		
pLNS	pN−	18 (60)	1288.72 ± 144.59	2	0.001^a^^,^^c^
	pN+	12 (40)	1658.25 ± 108.97		
pTNM	Early	15 (50)	1287.80 ± 136.28	24	<0.001^a^^,^^c^
	Advanced	15 (50)	1585.27 ± 197.40		
TB	Absent	15 (50)	1320.27 ± 181.16	43	0.004^a^^,^^c^
	Present	15 (50)	1552.80 ± 207.74		
PNI	Absent	19 (63)	1439.84 ± 257.09	103	0.949^a^
	Present	11 (37)	1430.82 ± 166.99		
Recurrence	Absent	20 (67)	1365.30 ± 183.74	45	0.016^a^^,^^c^
	Present	10 (33)	1579.00 ± 241.12		
Surgical Margins	Negative	25 (83)	1400.92 ± 226.06	25.5	0.039^a^^,^^c^
	Positive	5 (17)	1614.6 ± 114.45		
Survival	Dead	9 (30)	1599.22 ± 197.09	36	0.008^a^^,^^c^
	Alive	21 (70)	1366.81 ± 202.25		

a Mann–Whitney U.

b One-way Anova-Kruskal–Wallis test,

c Significance.

KWS, Kruskal—Wallis test statistics; cTNM, Clinical staging; pLNS, Pathological Lymph node status; pTNM, Pathological staging; PNI, Perineural Invasion; TB, Tumor budding.

Serum IFN-*γ* levels in OSCC patients showed significant associations with multiple CPC reflecting disease aggressiveness. Lower IFN-*γ* levels were observed in patients with multiple-site involvement, advanced clinical and pathological TNM stages, and greater TD (>1 cm) (all *p* < 0.05). Reduced IFN-*γ* was also significantly associated with poorer histological grade, LNM, TB, recurrence, positive surgical margins, and mortality (all *p* < 0.05) (Supplementary Table S2). Collectively, these findings indicate that diminished serum IFN-*γ* levels are linked to increased tumor burden, aggressive disease behaviour, and unfavorable prognosis in OSCC.

**Figure 2:**
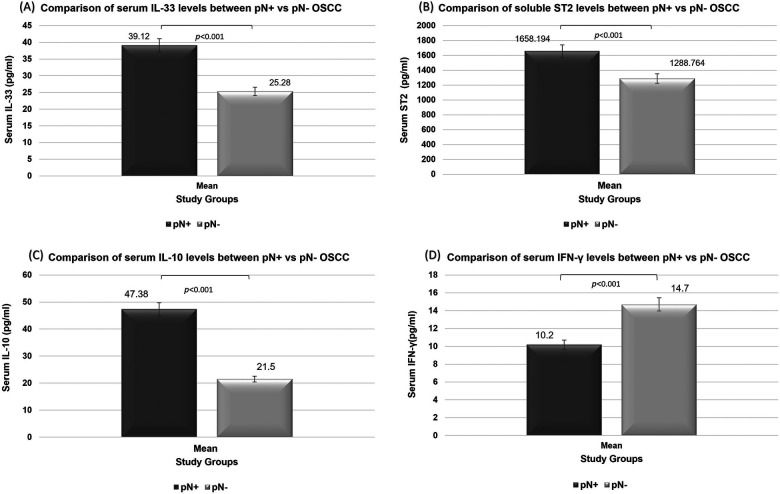
Comparison of serum levels of **(A)** IL-33, **(B)** soluble ST2, **(C)** IL-10, and **(D)** IFN-*γ* between metastatic (pN+) and non-metastatic (pN−) OSCC patients using the Mann–Whitney *U*-test.

Comparative analysis of serum cytokines demonstrated significant differences between metastatic (pN+) and non-metastatic (pN−) OSCC patients. Expression levels of IL-33, ST2, and IL-10 were significantly elevated in patients with pN+, whereas serum IFN-*γ* levels were significantly reduced in pN+ compared to pN− patients (all *p* < 0.001). Overall, these findings indicate that nodal involvement in OSCC is associated with a shift toward a tumor-promoting and immunosuppressive cytokine profile (Figure 2).

**Figure 3:**
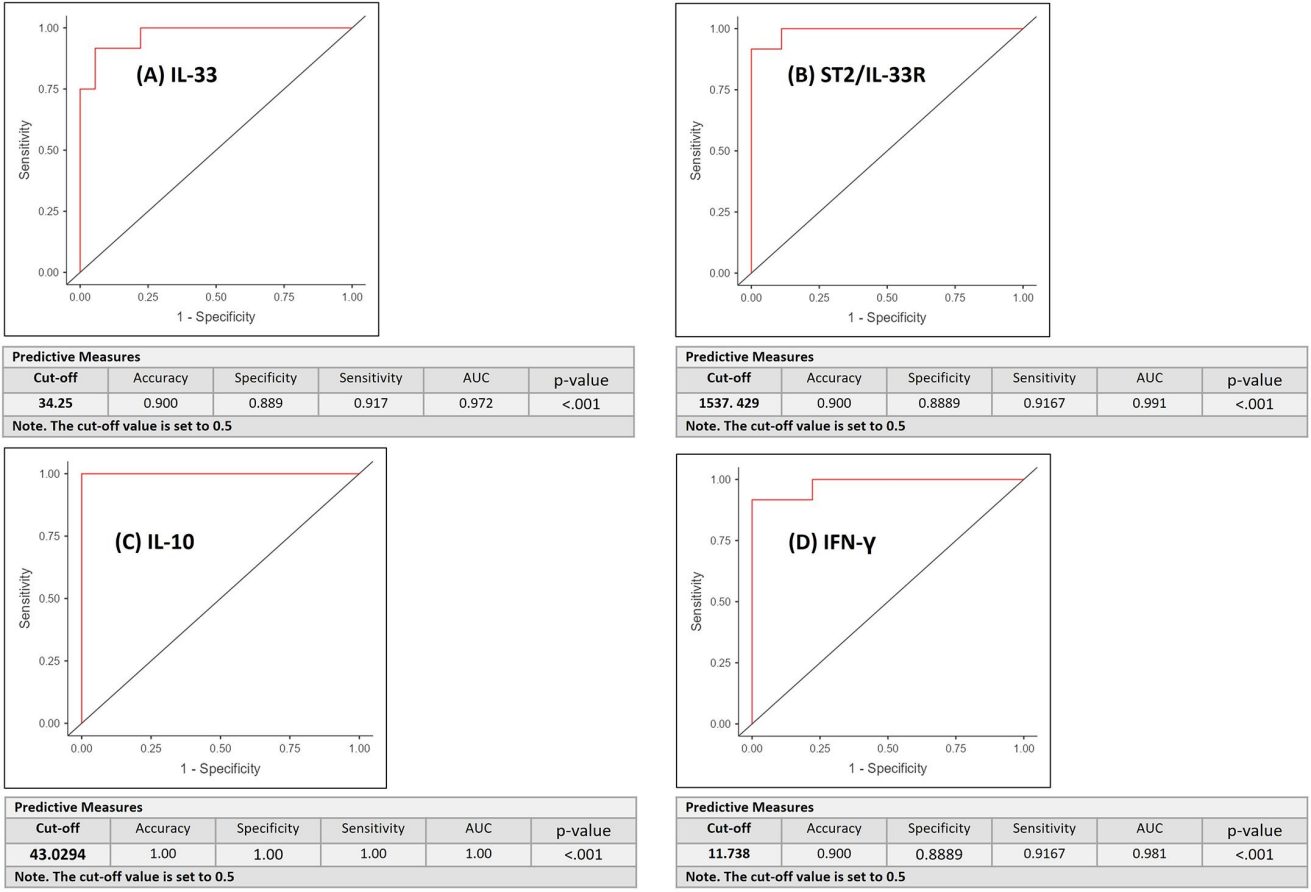
The ROC curves represent discriminatory ability of each mediator **(A)** IL-33, **(B)** ST2/ IL-33R, **(C)** IL-10, and **(D)** IFN-*γ* for differentiating metastatic (pN+) from non-metastatic (pN−) OSCC patients.

ROC curve analysis was performed to differentiate between pN+ and pN− OSCC patients based on serum cytokine levels. The curves represent discriminatory ability of each mediator (a) IL-33, (b) ST2, (c) IL-10, and (d) IFN-*γ* for differentiating pN+ from pN− OSCC patients (Figure 3). The AUC were 0.972, 0.991, 1.000, and 0.981, respectively. The optimal cut-off values yielded high discriminatory power, with accuracies ranging from 90% to 100%, sensitivities between 91.7% and 100%, and specificities between 88.9% and 100%, underscoring their strong predictive utility as serum biomarkers. IL-33, ST2, IL-10, and IFN-*γ* possess excellent discriminatory power (AUC >0.97 for all). DeLong's test revealed no statistically significant differences among the AUCs (overall *p* = 0.303). In terms of AUC none of the tested cytokine significantly outperformed the others.

BLR analyses assessed the ability of serum mediators to discriminate between pN+ and pN− OSCC patients (Supplementary Table S3). IL-33 and ST2 demonstrated excellent model fit and discrimination (AUC = 0.972 and 0.991, respectively; 90% accuracy), with higher levels showing borderline positive associations with LNM. IL-10 emerged as a strong positive predictor of LNM, with a highly significant model and perfect classification performance (AUC = 1.00), although the wide confidence interval suggested possible model instability due to small sample size. In contrast, IFN-*γ* was a significant negative predictor of nodal involvement, with higher levels markedly reducing LNM risk [Odds Ratio (OR) = 0.075] and excellent discriminative ability (AUC = 0.981). Overall, IL-10 showed the strongest positive predictive value for LNM, IL-33 and ST2 exhibited positive trends with high discriminatory accuracy. In contrast, IFN-*γ* demonstrated a pattern consistent with a marker of intact antitumor immunity, supporting the complementary utility of these mediators as biomarkers for lymph node risk stratification in OSCC.

**Figure 4:**
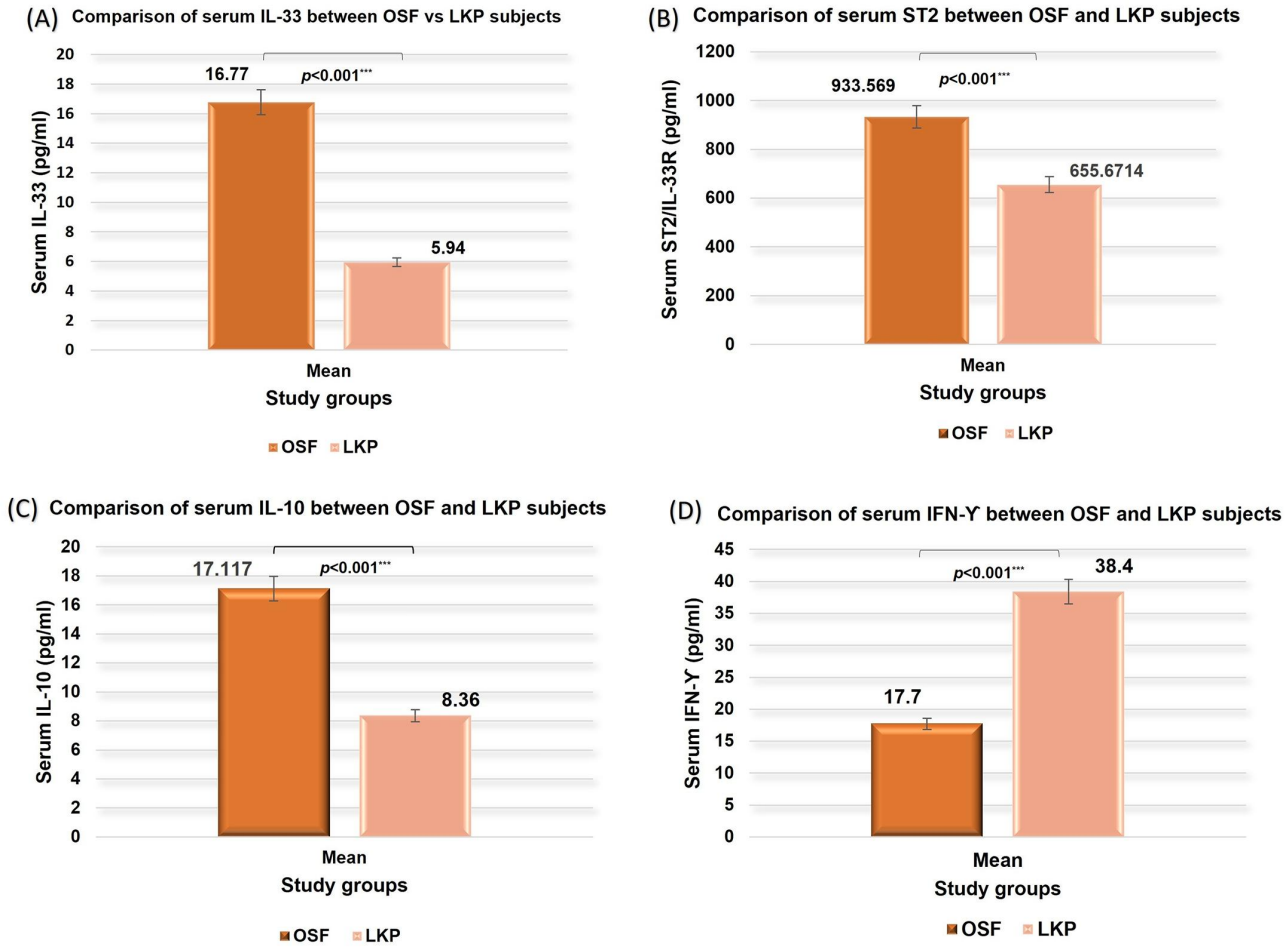
Comparison of serum levels of **(A)** IL-33, **(B)** ST2, **(C)** IL-10, and **(D)** IFN-*γ* between OSF and LKP subjects using the Mann–Whitney *U*-test.

Comparative analysis of serum cytokine levels revealed significant differences between OSF and LKP subjects across all immunomodulatory markers assessed. Serum IL-33, ST2, and IL-10 levels were significantly higher in OSF patients compared to those with LKP, whereas serum IFN-*γ* levels were significantly lower in the OSF group (all *p* < 0.001). These findings indicate a distinct immunological profile in OSF characterized by a shift toward a pro-tumorigenic and immunosuppressive cytokine milieu relative to LKP (Figure 4).

**Figure 5:**
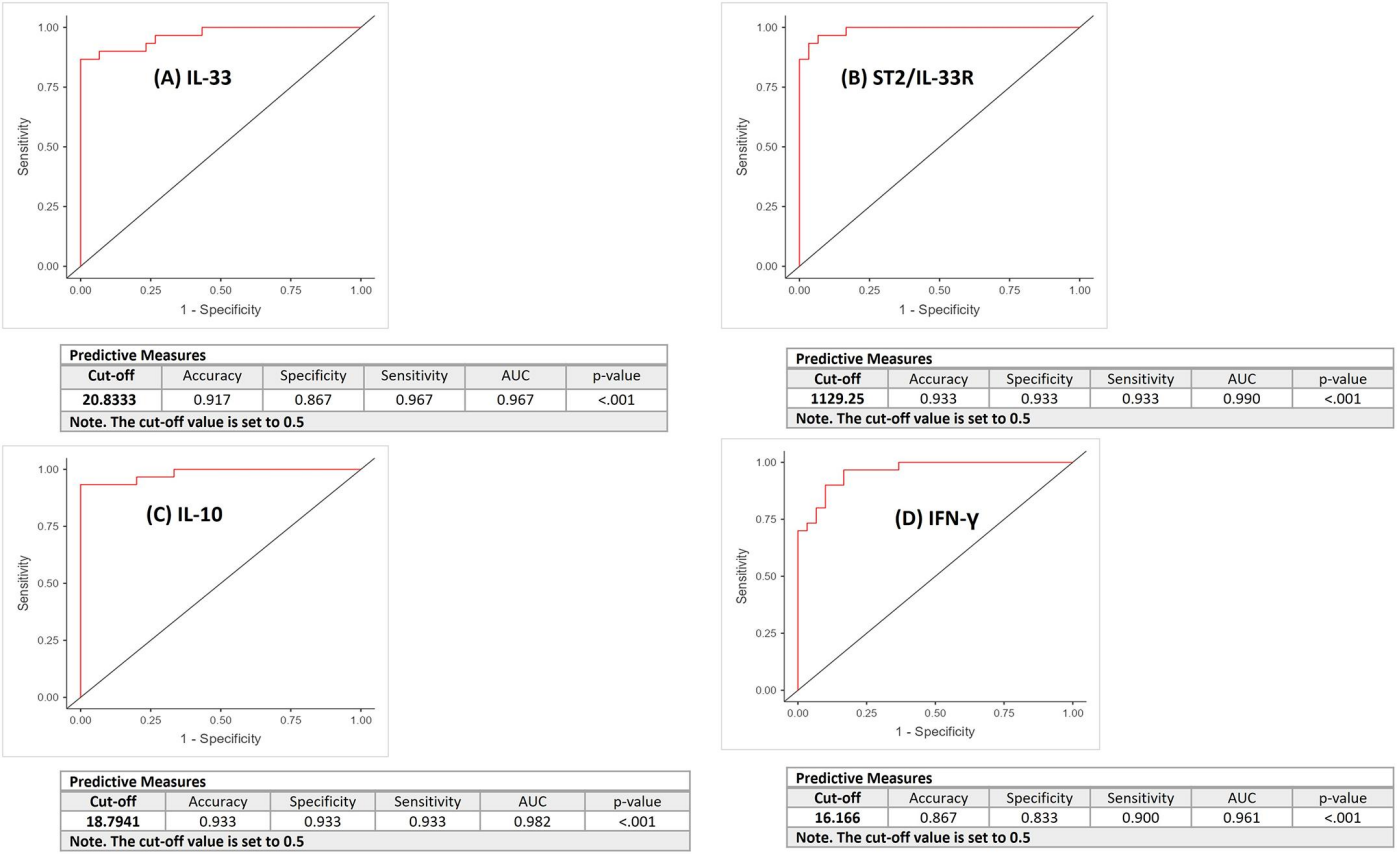
The ROC curves depict the diagnostic performance of each mediator **(A)** IL-33, **(B)** ST2/ IL-33R, **(C)** IL-10, and **(D)** IFN-*γ* for differentiating OSCC and OPMDs.

ROC analysis demonstrated excellent diagnostic performance of all four serum cytokines in differentiating OSCC from OPMDs. The AUCs were 0.967 for IL-33, 0.990 for ST2, 0.982 for IL-10, and 0.961 for IFN-*γ*, with all *p*-values < 0.001, indicating performance significantly better than chance. Optimal cut-off values yielded high diagnostic accuracy (86.7%–93.3%), sensitivities of 90.0%–96.7%, and specificities of 83.3%–93.3%. ST2 showed the highest AUC (0.990), followed by IL-10, IL-33, and IFN-*γ*; however, DeLong's test showed no significant differences between AUCs (overall *p* = 0.592), indicating comparable diagnostic potential among biomarkers. Thus, all four cytokines exhibit strong and similar distinguishing ability, and selection may depend on practical considerations such as assay availability, cost, or biological relevance (Figure 5).

BLR was also performed to evaluate the ability of serum cytokines to discriminate between OSCC and OPMDs subjects. IL-33, ST2, and IL-10 were positively associated with OSCC, while IFN-*γ* showed an inverse relationship (Table 3). Specifically, elevated IL-33 (OR = 1.55, 95% CI: 1.15–2.07, *p* = 0.004), ST2 (OR = 1.04, 95% CI: 1.00–1.08, *p* = 0.050), and IL-10 (OR = 3.26, 95% CI: 1.39–7.67, *p* = 0.007) significantly increased the odds of OSCC diagnosis. In contrast, higher IFN-*γ* levels were inversely associated with OSCC risk (OR = 0.41, 95% CI: 0.23–0.73, *p* = 0.002). Overall, serum IL-33, ST2, and IL-10 were positively associated with OSCC risk, whereas IFN-*γ* demonstrated an inverse association. All four cytokines showed high diagnostic performance, suggesting their potential utility in differentiating OSCC from OPMDs.

**Table 3:** Binary logistic regression analysis.

Model Fit Measures										
								Overall Model Test		
Model	Deviance	AIC	BIC	R²_McF_	R²_CS_	R²_N_	R²_T_	*χ*²	Df	*p*
1	27.4	31.4	35.6	0.671	0.605	0.807	0.715	55.8	1	<.001
Note. Models estimated using sample size of *N* = 60										
Model Coefficients—Diagnosis										
	95% CI								95% CI	
Predictor	Estimate	Lower	Upper	SE	Z	*p*	Odds ratio		Lower	Upper
Intercept	−8.897	−15.074	−2.721	3.151	−2.82	0.005	1.37e − 4		2.84e − 7	0.0658
**IL-33**	0.435	0.141	0.729	0.150	2.90	0.004	1.55		1.15	2.0732
Note. Estimates represent the log odds of “OSCC = 1” vs. “OPMDs = 0”										
Model Fit Measures										
								Overall Model Test		
Model	Deviance	AIC	BIC	R²_McF_	R²_CS_	R²_N_	R²_T_	χ²	Df	*p*
1	14.2	18.2	22.3	0.830	0.683	0.911	0.842	69.0	1	<.001
Note. Models estimated using sample size of *N* = 60										
Model Coefficients—Diagnosis										
	95% CI								95% CI	
Predictor	Estimate	Lower	Upper	SE	Z	*p*	Odds ratio		Lower	Upper
Intercept	−44.1728	−88.6	0.2294	22.6546	−1.95	0.051	6.55e − 20		3.41e − 39	1.26
**ST2**	0.0392	−3.38e − 5	0.0785	0.0200	1.96	0.050	1.04		1.000	1.08
Note. Estimates represent the log odds of “OSCC = 1” vs. “OPMDs = 0”										
Model Fit Measures										
								Overall Model Test		
Model	Deviance	AIC	BIC	R²_McF_	R²_CS_	R²_N_	R²_T_	χ²	Df	*p*
1	21.4	25.4	29.6	0.743	0.643	0.857	0.785	61.8	1	<.001
Note. Models estimated using sample size of *N* = 60										
Model Coefficients—Diagnosis										
	95% CI								95% CI	
Predictor	Estimate	Lower	Upper	SE	Z	*P*	Odds ratio		Lower	Upper
Intercept	−22.19	−38.227	−6.15	8.182	−2.71	0.007	2.30e − 10		2.50e − 17	0.00212
**IL-10**	1.18	0.328	2.04	0.436	2.71	0.007	3.26		1.39	7.67348
Note. Estimates represent the log odds of “OSCC Diagnosis = 1” vs. “OPMDs Diagnosis = 0”										
Model Fit Measures										
								Overall Model Test		
Model	Deviance	AIC	BIC	R²_McF_	R²_CS_	R²_N_	R²_T_	χ²	Df	*p*
1	30.2	34.2	38.4	0.637	0.586	0.782	0.668	53.0	1	<.001
Note. Models estimated using sample size of *N* = 60										
Model Coefficients—Diagnosis										
	95% CI								95% CI	
Predictor	Estimate	Lower	Upper	SE	Z	*p*	Odds ratio		Lower	Upper
Intercept	14.598	5.25	23.950	4.771	3.06	0.002	2.19e + 6		189.926	2.52e + 10
**IFN-*γ***	−0.888	−1.46	−0.315	0.292	−3.04	0.002	0.412		0.232	0.730

Estimates represent the log odds of “OSCC = 1” vs. “OPMDs = 0”.

## 4 Discussion

In this investigation, IL-33 levels were not detectable in all participants, particularly within the HC and OPMDs groups. This aligns with existing evidence showing that serum IL-33 is often low or undetectable in healthy individuals because its circulating concentrations typically remain below the detection limits of standard ELISA assays (38). Although IL-33 is constitutively expressed at low levels under steady-state conditions. When released into the extracellular space, IL-33 is rapidly inactivated through oxidation and proteolytic cleavage, resulting in a very short serum half-life. Consequently, circulating IL-33 remains low or undetectable. IL-33 predominantly resides in the nucleus, where IL-33_FL_ binds chromatin and suppresses inflammatory gene expression. It functions as an alarmin, released into the extracellular space mainly in response to tissue injury, necrosis, mechanical stress, or cell damage. In the absence of triggers, IL-33 remains sequestered in the nucleus to maintain homeostasis, resulting in very low basal serum levels (4). Consequently, several studies have reported that only a fraction of HC exhibit detectable serum IL-33, with detectability varying widely depending on assay sensitivity, sample handling, and population characteristics (38, 39).

In contrast, in cancer, continuous cellular stress, necrosis, and tissue disruption within the TME led to sustained release of IL-33 from tumor cells, stromal cells, and infiltrating immune cells. This persistent release can maintain detectable systemic IL-33 levels despite its rapid inactivation. In this investigation all OSCC participants exhibited measurable IL-33 levels. In several cancers, circulating IL-33 becomes detectable—and often increased—possibly reflecting release of IL-33 from tumor cells, stromal cells (e.g., Carcinoma associated fibroblast), or damaged tissue within the TME (33, 34). The degree of detection and the measured concentrations vary considerably depending on cancer type, stage, assay sensitivity, sample handling, and possibly geographic/population differences. The reviews and meta-analysis noted substantial heterogeneity across studies (10, 11, 40). In the context of HNSCC—most studies focus on tissue expression (IHC, gene expression) rather than serum IL-33. A recent analysis of the included studies showed that IL-33 is expressed in the tumor tissue of HNSCC, predominantly within the stromal compartment. Enhanced IL-33 expression was particularly observed in CAFs, and in some studies its overexpression correlated with IL-33 expression in adjacent tumor cells. Increased stromal IL-33 was associated with unfavorable clinical outcomes, including poor nodal metastasis-free survival, indicating an adverse prognosis. The IL-33–rich TME was also linked to increased ST2-expressing regulatory T cells (Tregs), suggesting that tumor tissue IL-33 may promote an immunosuppressive microenvironment that supports tumor progression (34).

Soluble ST2 functions as a sequestering receptor for IL-33, modulating the cytokine's availability and biological activity (33). Through this regulatory role, soluble ST2 attains biological significance in its own right, rather than acting merely as a passive biomarker (4, 41). In this analytical study soluble ST2 were measurable in all study groups. Investigations on breast cancer (BC) (42, 43). gastric cancer (GC) (44), non-small cell lung cancer (NSCLC) (45) confirms that soluble ST2 is quantifiable in normal (healthy) sera—establishing a baseline against which disease-associated changes can be measured. In different malignancies (BC, GC, NSCLC), soluble ST2 levels are often higher in cancer patients compared to HC, suggesting soluble ST2 may reflect tumor-associated processes like inflammation, tissue remodeling and immune dysregulation (33, 42–45).

IL-33 and IL-10 are functionally interconnected within the inflammatory network, as IL-33 can induce downstream IL-10 production, thereby promoting an anti-inflammatory or regulatory IR. Within the TME, IL-33–mediated signaling often drives the expansion of regulatory immune cell subsets, many of which secrete IL-10 (13, 17). Under physiological conditions, IL-10 is produced at very low levels and is often undetectable in circulation, a homeostatic mechanism that prevents unwarranted inflammatory activation (46, 47). In agreement with this, the present study found that the HC showed the lowest serum IL-10 concentrations among all cohorts, with measurable IL-10 detected in only 43% of HC. In contrast, elevated circulating IL-10 has been widely reported across multiple malignancies—including NSCLC (48), BC (49), OSCC, and HNSCC (50–52)—where it is associated with tumor-driven immunosuppression and disease progression (48–50, 52). Consistent with this pattern, all subjects in the OPMDs and OSCC groups in the current study exhibited measurable IL-10 levels, supporting previous findings that IL-10 is upregulated in premalignant and malignant oral lesions (53, 54).

In the present study, all participants—including HC, subjects with OPMDs, and OSCC patients—exhibited measurable serum IFN-*γ* concentrations. However, a progressive decline was evident across the disease spectrum, with OSCC patients displaying the lowest levels. Healthy individuals showed relatively higher basal IFN-*γ*, consistent with intact immune surveillance and the central role of IFN-*γ* in driving cell-mediated antitumor responses (10). These findings align with earlier evidence demonstrating markedly reduced circulating IFN-*γ* in HNSCC compared with healthy individuals, indicative of disease-associated immune suppression. Such diminished IFN-*γ* production reflects impaired Th1-mediated antitumor immunity, a key feature of tumor immune evasion (55, 56).

In this investigation serum IL-33 levels showed a stepwise rise from health to disease, with the lowest concentrations in HC, higher levels in OPMDs, and the highest levels in OSCC. This progressive elevation of IL-33 strongly suggests its potential utility as a biomarker of MT and disease progression. This study represents, to the best of our knowledge, the first evaluation of serum IL-33 in OSCC. The expression of serum IL-33 has been examined across numerous cancer types, including NSCLC (9, 28, 29, 45), Hepatocellular carcinoma (HCC) (12, 30, 40) BC (42, 43), Colorectal cancer (CRC) (31, 32), GC (9, 44), Prostate cancer (PC) (57), esophageal adenocarcinoma (EAC) (58), renal cell carcinoma (RCC) (59), and endometrial cancer (EC) (60). A recent review encompassing 38 studies on serum IL-33 in various cancers found elevated levels in 26 of them (68%) (33). A meta-analysis examined the relationship between circulating IL-33 and tumor progression across multiple malignancies. It found that elevated circulating IL-33 was significantly associated with tumor presence, supporting a potential role for IL-33 in cancer development (10).

Elevated IL-33 levels reported across multiple malignancies suggest its active involvement in tumor-promoting inflammation, fostering Th2-skewed immunity and supporting tumor cell survival. Detectable circulating IL-33 in cancer patients likely reflects ongoing cellular stress, necrosis, or tissue disruption associated with tumor progression. Once released, circulating IL-33 engages the IL-33/ST2 signaling cascade on immune and stromal cells, contributing to a pro-tumorigenic microenvironment. This occurs through promotion of angiogenesis and vascular permeability, recruitment and polarization of immunosuppressive cell populations (Tregs, M2-macrophages, MDSCs), and, in some contexts, direct enhancement of tumor-cell proliferation, invasion, and metastatic potential. Consequently, systemic IL-33 levels may serve as a surrogate inflammatory signature that mirrors the underlying tumor biology and immunomodulatory dynamics within the TME (4, 13, 17, 19, 33).

Soluble ST2, a ligand trap that sequesters IL-33, acts as a central regulator of its extracellular presence and downstream effects (33). Research on soluble ST2 in cancer patients is still limited and shows conflicting results (15, 28, 42). Among studies assessing soluble ST2 in cancer, the majority examined HCC (12, 40) followed by BC (42, 43), pancreatic ductal adenocarcinoma (PDAC) (61), CRC (62), NSCLC (29, 45), HNSCC (63–65), GC (44), and EAC (58). A recent review encompassing 22 studies on soluble ST2 in various cancers found 55% (12 out of 22) reported increased soluble ST2 levels (33). Elevated ST2 concentrations have been documented across multiple malignancies, including HCC (12, 40), BC (42, 43) PDAC (61), NSCLC (29), HNSCC (65) and GC (44).

Similarly, this study demonstrated significantly elevated serum ST2 levels in OSCC compared with OPMDs and HC, demonstrating a gradient increase with disease progression and tumorigenic advancement. Emerging findings demonstrate that the relative expression of ST2L and soluble ST2 directly affects the TME and contributes to the progression of multiple cancer types (66). The functional impact of IL-33/ST2 signaling in malignancy largely depends on the relative expression of ST2L and its soluble counterpart, sST2. Overexpression ST2L enhances IL-33–driven activation of immune and stromal cells, leading either to anti-tumor responses—via cytotoxic T-cell and NK-cell activation—or to pro-tumor effects such as chronic inflammation, angiogenesis, and M2 macrophage polarization, in a manner dictated by TME. In contrast, higher sST2 levels serve as a decoy receptor, capturing IL-33 and suppressing ST2L-dependent signaling. This shift may suppress IL-33–driven anti-tumor IR or, in certain settings, restrain IL-33–induced pro-tumor inflammation. Consequently, the balance between ST2L and sST2 expression is a key determinant of whether the IL-33/ST2 axis ultimately exerts tumor-promoting or tumor-suppressive effects.(4, 41, 62, 67).

Adding to this complexity, recent reviews on soluble cytokine receptors note “discordant” evidence regarding soluble ST2: while many studies support its classical decoy role, others report agonist-like or context-dependent functions (68). Besides acting as a promiscuous receptor, emerging evidence suggests that soluble ST2 may have additional biological functions. It has been proposed that soluble ST2 might behave as an extracellular ligand capable of interacting with an as-yet unidentified receptor, implying a secondary role as an inflammatory mediator or even a growth-promoting factor rather than merely a neutralizing receptor isoform (19). In recent years, soluble ST2 has gained recognition as a pro-inflammatory marker, with its production being upregulated by classical cytokines such as IL-1β and TNF-α (16).

The IL-33/ST2 pathway activates downstream signaling that induces Th2 cytokines and immunosuppressive mediators like IL-10 and TGF-*β*, that contribute to immune regulation and tumor immune evasion (33). Serum IL-10 have been extensively studied in solid tumors and HM (69). IL-10, a pleiotropic anti-inflammatory cytokine, contributes to immunosuppression and facilitates tumor immune escape (70) Elevated serum IL-10 has been documented in BC (71), HCC (72), NSCLC (73), and HNSCC (50–54, 74–76). Multiple investigators—have consistently reported significantly higher serum IL-10 levels in HNSCC patients compared with HC (6, 25, 50–54, 74, 76) In the present study, IL-10 demonstrated a steady increase across groups, being lowest in HC, intermediate in patients with OPMDs, and highest in those with OSCC. This graded rise reinforces the relevance of IL-10 in reflecting disease progression.

IFN-*γ*, an essential proinflammatory cytokine functioning in both innate and adaptive immunity, contributes to tumor surveillance and mediates anti-tumor activity by promoting apoptosis (55, 77). Reduced serum IFN-*γ* levels have been reported in both NSCLC (29) and HNSCC (25, 55). In HNSCC, this decline has been consistently documented by several researchers, who observed significantly lower IFN-Ƴ levels in patients compared with HC (5, 25, 55, 74, 78). In the present study, IFN-*γ* concentrations differed significantly among groups, being highest in OPMDs (especially in LKP group), intermediate in HC, and lowest in OSCC. These findings indicate that IFN-*γ* expression is relatively preserved in OPMDs but declines sharply with MT, reflecting impaired host IR in OSCC.

In the present study, elevated serum IL-33 in OSCC were significantly associated with CPC of prognostic relevance, like TS, stage, grade, and LNM. Higher concentrations appear to reflect tumor burden and advancing disease stage, linking IL-33 expression with aggressive tumor characteristics and poorer prognosis. Comparable significant clinicopathological correlations have been reported in NSCLC (9, 29), HCC (12, 30, 40), GC (8, 44), CRC (31, 32), BC (79) PC (57), EAC (58), RCC (59), and EC (60). Elevated serum IL-33 observed in numerous malignancies underscores its role as a pathological “alarmin”—released during tumor-associated tissue stress or damage—rather than a constitutive secreted cytokine. Its measurable serum levels have been shown to correlate with clinical severity—including advanced stage, local invasion, and metastatic spread—indicating that serum IL-33 may function as a valuable non-invasive indicator for cancer detection, prognostication, and disease monitoring. Mechanistically, IL-33 within the TME may drive tumor progression by reshaping IR and fostering either immunosuppression or immune evasion (8–10, 17, 29, 30, 40, 79).

In this analysis, observations revealed that OSCC patients with larger tumors and advanced TNM stage exhibited markedly elevated soluble ST2 levels. Similarly, higher concentrations were associated with greater TV and increased cranio-caudal depth. Significant elevations were also observed in cases with poor differentiation and with LNM. Moreover, the presence of TB, positive surgical margins, with recurrence and reduced survival correlated with increased ST2 expression. Overall, in OSCC, elevated soluble ST2 showed strong associations with multiple clinicopathological parameters of prognostic relevance. These results suggest that ST2 may serve as a valuable indicator of tumor aggressiveness and poor prognosis in OSCC. Comparable significant clinicopathologic relations have been observed in HCC (12), BC (43), CRC (62), and GC (44). Clinical studies have shown that higher serum ST2 levels often correlate with advanced tumor stage, metastasis, poor differentiation, and worse prognosis, suggesting that ST2 may facilitate tumor progression and immune evasion (12, 43, 44, 62).

Notably, no prior studies have reported serum IL-33 or soluble ST2 levels in OSF or LKP, making these findings novel in the existing literature. The comparison of serum cytokine profiles between OSF and LKP subjects revealed distinct immuno-inflammatory patterns across all analytes assessed. Patients with OSF showed significantly elevated circulating levels of IL-33, soluble ST2, and IL-10, alongside markedly reduced IFN-*γ* concentrations, reflecting a shift toward a pro-fibrotic and immunosuppressive cytokine milieu, whereas LKP demonstrated a comparatively more pro-inflammatory profile. These serum findings are consistent with broader principles of fibrotic disease, in which chronic tissue injury and persistent inflammation lead to excessive ECM accumulation and progressive scarring. IL-33—well recognized for its dual role as both a cytokine and nuclear alarmin—has emerged important modulator of fibrosis, with the IL-33/ST2 cascade exerting context-dependent effects across fibrotic disorders, promoting inflammation and fibrosis in most conditions while exerting anti-fibrotic effects in others (80). Converging evidence supports its pathogenic role in OSF (81, 82).

Finally, regarding the diagnostic performance of these immunomodulatory mediators, ROC curve analysis demonstrated that IL-33, ST2, IL-10, and IFN-*γ* each showed excellent discriminatory ability between OSCC and OPMDs. Similar ROC analyses of immune mediators were conducted by Hu et al., (9) in NSCLC, Bergis et al., (44) in GC and by Shen et al., (72) in HCC to discriminate between the two groups. In this investigation, logistic regression further indicated that IL-33, ST2, and IL-10 were positively associated with OSCC, whereas IFN-*γ* showed an inverse association. Collectively, these findings underscore the clinical value of cytokine profiling: IL-33, ST2, and IL-10 reflect tumor-promoting immune alterations, while IFN-*γ* aligns with its well-established anti-tumor role. The seemingly robust performance of this study's integrated cytokine panel highlights its potential as a non-invasive tool for diagnostic distinction and risk stratification of OSCC.

In OSCC, the Th1/Th2 shift is multifactorial and mediated by a complex interplay of cytokines and immune regulatory pathways. Cytokines such as IL-4, IL-5, IL-10, IL-13, IL-12, and IFN, together with signals from antigen-presenting cells and Tregs, influence T-helper cell polarization within the TME. Additional factors, including chronic inflammation, hypoxia, and tumor-derived mediators, may further skew IR toward a tumor-promoting state. Within this context, the IL-33/ST2 axis can contribute to Th2-skewed IR and enhance regulatory immune pathways. In the present study, IL-33, ST2, IL-10, and IFN-*γ* represent mediators operating through complementary immunological mechanisms. Although each cytokine demonstrated comparable diagnostic performance individually, their combined evaluation as a composite biomarker panel may provide greater biological insight and improved clinical utility for discriminating LNS and risk stratification than individual biomarker analysis.

This study is novel in simultaneously evaluating IL-33, soluble ST2, IL-10, and IFN-*γ* in HC, OPMDs, and OSCC patients, providing an integrated view of their immunoregulatory interplay across the disease spectrum. It is among the first to examine the concurrent serum profiles of IL-33 and soluble ST2 in OPMDs and OSCC, thereby revealing a coordinated cytokine network that drives malignant progression. Importantly, the study also highlights, probably for the first time, the diagnostic value of these immunomodulators—especially IL-10 and IFN-*γ*—distinguishing OSCC from OPMDs.

The key strengths of this investigation include its novel focus, rigorous statistical analysis, and patient data collection in a dual-center setting, which enhances the reliability of the findings. Importantly, the combined assessment of IL-33, soluble ST2, IL-10, and IFN-*γ* appears to provide greater sensitivity and specificity than evaluating individual biomarkers alone. Evidence suggests that a panel approach could improve prediction of malignant transformation, invasiveness, progression, and outcomes.

This study has certain limitations. The IL-33/ST2 pathway exhibits highly context-dependent and microenvironment-specific functions that may vary across different stages of tumor progression. The inherent biological complexity, together with tumor heterogeneity, makes interpreting circulating levels of IL-33, soluble ST2, IL-10, and IFN-*γ* challenging. Moreover, serum cytokine profiles can be influenced by unrecognized inflammation, allergies, or subclinical conditions, which may compromise specificity. Although strict selection criteria were applied, unmeasured confounders—such as diet, obesity, and periodontal status—may still have affected cytokine concentrations. Technical challenges in cytokine detection further limit interpretability. Many cytokines circulate bound to soluble receptors, carrier proteins or inhibitors making them difficult to measure accurately by ELISA or radioimmunoassay. Others are produced locally at the disease site and degrade rapidly due to their short half-life, rendering them undetectable in serum. Additionally, standard immunoassays cannot differentiate between full-length and cleaved IL-33 isoforms, despite the fact that isoform size strongly affects bioactivity. For future studies, Western blotting or advanced proteomic approaches are recommended to characterize IL-33 isoforms associated with OSCC. Measurements of serum cytokine levels can also be performed using mass spectrometry–based proteomic approaches. Compared with ELISA, mass spectrometry provides higher specificity and enables simultaneous quantification of multiple cytokines with greater precision. It can also distinguish protein isoforms, detect post-translational modifications, and reduce antibody-related cross-reactivity. Thus, its application in future studies could help validate ELISA findings and overcome some methodological limitations, strengthening the reliability of cytokine biomarker research.

Owing to the study's cross-sectional design, modest sample size, and convenience sampling strategy, the generalizability of the findings is limited and warrants cautious interpretation. Although participants were recruited from two centres, inclusion based on availability and predefined criteria rather than random sampling may have reduced the representativeness of the study population and introduced potential selection bias, which could lead to overestimation of ROC-derived cut-off values and other diagnostic performance metrics. In addition, cytokine levels were measured at a single time point; therefore, temporal variations or disease-progression–related changes could not be evaluated. Longitudinal studies are needed to assess dynamic alterations in IL-33, ST2, IL-10, and IFN-*γ* during the transition from OPMDs to OSCC. Furthermore, the relationship between serum and salivary IL-33 remains unexplored and warrants further investigation.

Future research should explore the relationship between these cytokines and established tumor markers while further investigating the IL-33/ST2 axis as both a prognostic biomarker and a potential therapeutic target. Comprehensive evaluation of IL-33 and ST2 across tumor tissues and body fluids, together with programmed death-ligand 1 (PD-L1) profiling, may provide deeper insight into their collective role in HNSCC biology. From a therapeutic perspective, selective targeting of the IL-33/ST2 signaling pathway represents a promising avenue. The IL-33/ST2 cascade may synergize with immune checkpoint inhibitors (ICIs), potentially overcoming therapeutic resistance by reducing immunosuppressive cell populations and enhancing effector T-cell responses. Consequently, combining IL-33/ST2-directed strategies with ICIs may improve treatment outcomes.

In addition, mechanistic *in-vitro* studies using healthy, premalignant, and malignant oral cell lines would help validate the biological role of these cytokines in disease progression. Cytokine levels could initially be quantified in the conditioned media of these cell lines to compare secretion patterns, followed by treatment with individual cytokines or their combinations to assess functional effects through proliferation, invasion, and wound-healing assays. Such experimental approaches, together with robust clinical trials and multi-center studies employing standardized assays and longitudinal sampling, are essential to clarify the diagnostic, prognostic, and therapeutic utility of IL-33 and soluble ST2 in OPMDs, OSCC, and the broader management of HNSCC.

## 5 Conclusion

A progressive increase in IL-33, soluble ST2, and IL-10 together with reduced IFN-*γ* indicates a shift toward pro-tumorigenic inflammation and immune evasion, supporting their potential utility as biomarkers of malignant progression. Cytokine interaction patterns differed across groups: in OPMDs and OSCC, IL-33, soluble ST2, and IL-10 formed a strongly interconnected, mutually reinforcing network, while IFN-*γ* consistently demonstrated inverse associations with all three, highlighting its antagonistic role in tumor-promoting inflammation. These correlation patterns suggest that IL-33, soluble ST2, and IL-10 form a synergistic network that counteracts IFN-*γ*, a relationship that intensifies from OPMDs to OSCC, highlighting their role in promoting neoplastic transformation, driving tumor progression, and impairing antitumor immunity. Serum IL-33, soluble ST2, IL-10, and IFN-*γ* also showed strong associations with prognostically relevant clinicopathologic parameters, further emphasizing their potential as biomarkers in OSCC. Diagnostic analyses showed that IL-33, soluble ST2, IL-10, and IFN-*γ* each possessed notable discriminatory ability for predicting LNM, with IL-10 being the strongest positive predictor and IFN-*γ* demonstrating a favorable prognostic influence. These mediators also effectively distinguished OSCC from OPMDs, with elevated IL-33, soluble ST2, and IL-10 indicating higher disease risk, while higher IFN-*γ* levels suggested reduced risk.

## Data Availability

The datasets presented in this study can be found in online repositories. The names of the repository/repositories and accession number(s) can be found in the article/Supplementary Material.
