# Supplementary material for: Immunomodulatory mediators IL-33, soluble ST2, IL-10, IFN-γ in the serum of patients with oral potentially malignant disorders and oral squamous cell carcinoma
Source: Front Oral Health. 2026 Apr 20;7:1793916. doi: 10.3389/froh.2026.1793916 (PMC13136695; doi:10.3389/froh.2026.1793916)
Supplement: Supplementary file 1 [file Table1.docx]

| Table 1. The association between serum IL-10 levels and the CPC of OSCC | | | | | |
| --- | --- | --- | --- | --- | --- |
| Parameters | **Category** | **n%** | **Serum IL-10**  **Mean ± SD** | **U – statistic** | ***p*-value** |
| Age | ≤45  >45 | 4(13)  26(87) | 35.5±12.26  31.35±13.52 | 35.5 | 0.311^#^ |
| Sex | Male  Female | 22(73)  8(27) | 31.27±13.35  33.63±13.65 | 81.5 | 0.759^#^ |
| Habits | Absent  Present | 7(23)  23(77) | 28.29±11.84  33.0±13.69 | 60.5 | 0.324^#^ |
| Site of the lesion | Buccal Mucosa  Tongue  Gingiva & others | 8(27)  12(40)  10(33) | 28.25±12.43  28.08±11.02  39.40±14.23 | 2.344 **^KWS^** | 0.130^@^ |
| Extension of the lesion | Single  Multiple | 21(70)  9(30) | 29.43±12.28  37.67±14.30 | 79 | 0.481^#^ |
| Nature of the lesion | Exophytic  Endophytic | 14(47)  16(53) | 34.36±13.18  29.75±13.32 | 89.5 | 0.347^#^ |
| Associated with OPMD | Absent  Present | 22(73)  8(27) | 31.5±12.79  33.0±15.27 | 78 | 0.637^#^ |
| Tumor Size | ≤4cms  >4cms | 22(73)  8(27) | 29.09±12.37  39.63±13.17 | 66 | 0.299^#^ |
| cTNM | Early (I&II)  Advanced (III & IV) | 11(37)  19(63) | 22.09±3.08  37.58±13.57 | 49 | 0.016^#*^ |
| Tumor Volume | ≤5cm^3^  >5cm^3^ | 17(57)  13(43) | 26.35±10.06  39.15±13.71 | 65.5 | 0.058^#^ |
| Depth | ≤ 1cm  >1cm | 17(57)  13(43) | 26.18±10.15  39.38±13.38 | 59.5 | 0.032^#*^ |
| Grade | Well-Moderately differentiated  Moderate-Poorly differentiated | 18(60)  12 (40) | 27.50±11.15  38.50±13.80 | 65.5 | 0.07^#^ |
| pLNS | pN-  pN+ | 18(60)  12(40) | 21.56±2.77  47.42±3.55 | 0 | <0.001^#*^ |
| pTNM | Early  Advanced | 15(50)  15(50) | 22.07±2.76  41.73±12.18 | 40.5 | 0.003^#*^ |
| TB | Absent  Present | 15(50)  15(50) | 25.2±10.57  38.60±12.45 | 52.5 | 0.012^#*^ |
| PNI | Absent  Present | 19(63)  11(37) | 32.47±14.21  30.91±11.97 | 100.5 | 0.863^#^ |
| Recurrence | Absent  Present | 20(67)  10(33) | 28.0±11.37  39.7±13.80 | 59.5 | 0.073^#^ |
| Surgical Margins | Negative  Positive | 25(83)  5(17) | 28.84±12.35  47.2±1.924 | 15.5 | 0.009^#*^ |
| Survival | Dead  Alive | 9(30)  21(70) | 40.89±12.72  28.05±11.72 | 56 | 0.08^#^ |
| ^#^ Mann-Whitney U; ^@^One-way Anova-Kruskal-Wallis test; * Significance; ^KWS^ Kruskal – Wallis test statistics; cTNM, Clinical staging; pLNS, Pathological Lymph node status; pTNM, Pathological staging; PNI, Perineural Invasion; TB, Tumor budding. | | | | | |

| Table 2. The association between serum IFN-Ƴ levels and the CPC of OSCC | | | | | |
| --- | --- | --- | --- | --- | --- |
| Parameters | **Category** | **n%** | **Serum IFN-Ƴ**  **Mean ± SD** | **U -statistic** | ***p*-value** |
| Age | ≤45  >45 | 4(13)  26(87) | 12.50±3.51  12.92±2.69 | 46 | 0.711^#^ |
| Sex | Male  Female | 22(73)  8(27) | 12.86±2.56  12.88±3.39 | 86.5 | 0.943^#^ |
| Habits | Absent  Present | 7(23)  23(77) | 13.86±2.98  12.57±2.67 | 61.5 | 0.346^#^ |
| Site of the lesion | Buccal Mucosa  Tongue  Gingiva & others | 8(27)  12(40)  10(33) | 13.12±2.74  13.75±2.66  11.60±2.63 | 3.613^KWS^ | 0.164^@^ |
| Extension of the lesion | Single  Multiple | 21(70)  9(30) | 13.62±2.61  11.11±2.31 | 39.5 | 0.012^#*^ |
| Nature of the lesion | Exophytic  Endophytic | 14(47)  16(53) | 12.64±2.97  13.06±2.62 | 104.5 | 0.753^#^ |
| Associated with OPMD | Absent  Present | 22(73)  8(27) | 13.09±2.77  12.25±2.76 | 72.5 | 0.462^#^ |
| Tumor Size | ≤4cms  >4cms | 22(73)  8(27) | 13.32±2.81  11.63±2.26 | 56.5 | 0.135^#^ |
| cTNM | Early (I&II)  Advanced (III & IV) | 11(37)  19(63) | 15.27±2.00  11.47±2.09 | 24.5 | <0.001^#*^ |
| Tumor Volume | ≤5cm^3^  >5cm^3^ | 17(57)  13(43) | 13.71±2.77  11.77±2.38 | 64.5 | 0.052^#^ |
| Depth | ≤ 1cm  >1cm | 17(57)  13(43) | 13.88±2.75  11.54±2.18 | 57 | 0.024^#*^ |
| Grade | Well-Moderately differentiated  Moderate-Poorly differentiated | 18(60)  12 (40) | 14.06±2.64  11.08±1.83 | 41 | 0.004^#*^ |
| pLNS | pN-  pN+ | 18(60)  12(40) | 14.61±2.09  10.25±0.86 | 6 | <0.001^#*^ |
| pTNM | Early  Advanced | 15(50)  15(50) | 14.80±2.04  10.93±1.87 | 19.5 | <0.001^#*^ |
| TB | Absent  Present | 15(50)  15(50) | 14.00±2.66  11.73±2.37 | 62 | 0.034^#*^ |
| PNI | Absent  Present | 19(63)  11(37) | 12.68±2.70  13.18±2.92 | 95 | 0.679^#^ |
| Recurrence | Absent  Present | 20(67)  10(33) | 13.65±2.81  11.30±1.88 | 51.5 | 0.031^#*^ |
| Surgical Margins | Negative  Positive | 25(83)  5(17) | 13.48±2.58  9.80±0.837 | 10 | 0.003^#*^ |
| Survival | Dead  Alive | 9(30)  21(70) | 10.78±2.16  13.76±2.50 | 32.5 | 0.005^#*^ |
| ^#^ Mann-Whitney U; ^@^One-way Anova-Kruskal-Wallis test; * Significance; ^KWS^ Kruskal – Wallis test statistics; cTNM, Clinical staging; pLNS, Pathological Lymph node status; pTNM, Pathological staging; PNI, Perineural Invasion; TB, Tumor budding. | | | | | |


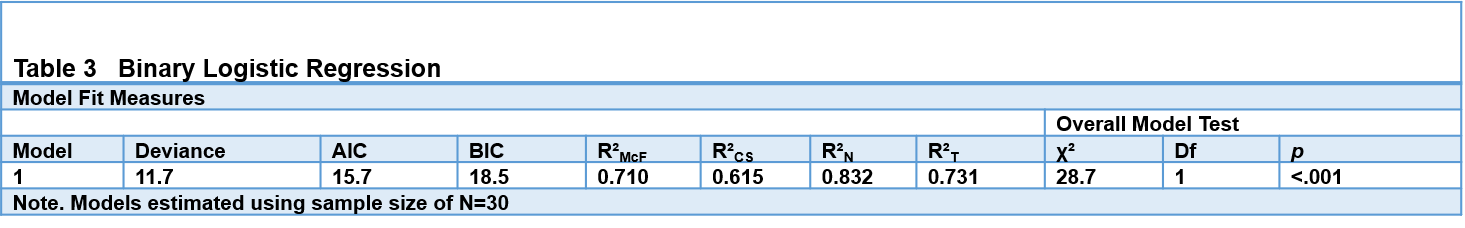

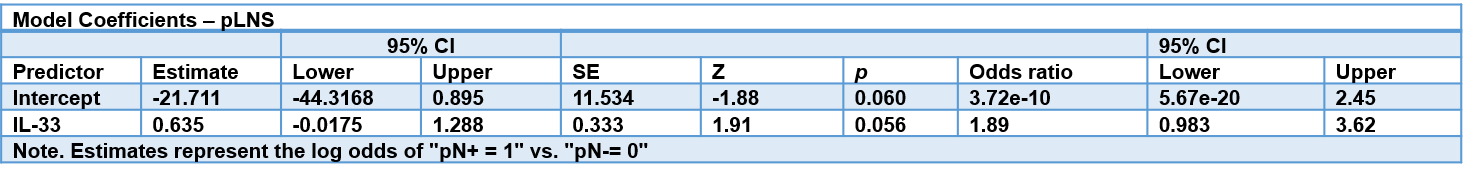

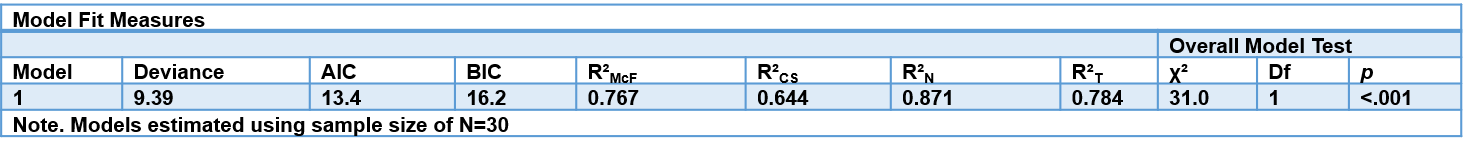

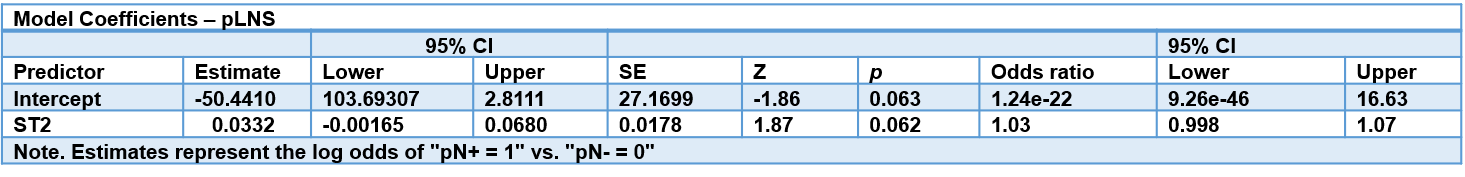

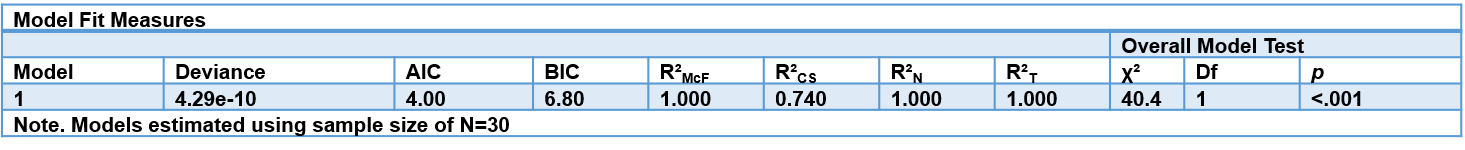

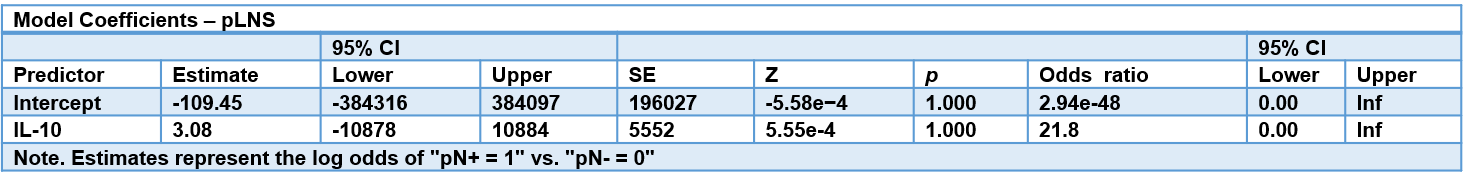

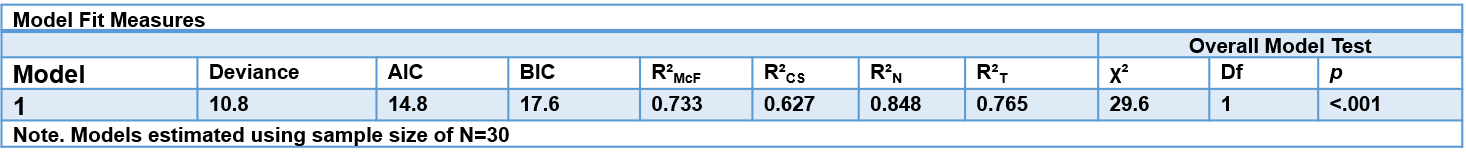

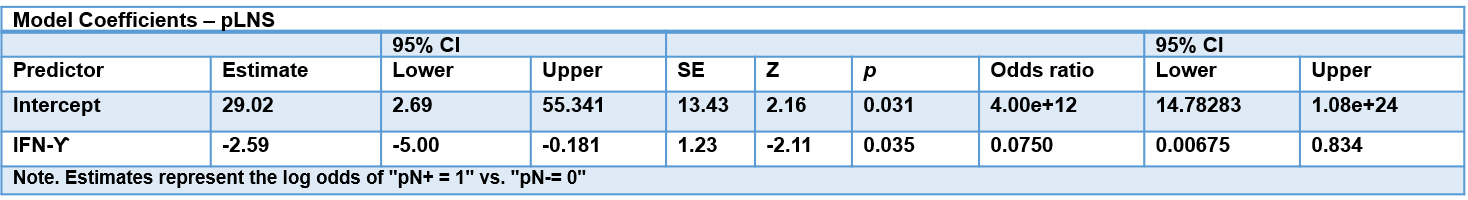


| Table 4. The association between serum IL-33, ST2, IL-10, IFN-γ levels and the CPC of OPMDs | | | | | |
| --- | --- | --- | --- | --- | --- |
| Parameters | **Category** | **n%** | **IL-33 (Mean ± SD)** | **U statistic** | ***p*-value** |
| Age | ≤45  >45 | 8(27)  22(73) | 13.82±6.79  10.46±7.11 | 60.5 | 0.196^#^ |
| Sex | Male  Female | 24(80)  6(20) | 10.7±6.36  13.9±9.73 | 59 | 0.499^#^ |
| Habits | Absent  Present | 5(17)  25(83) | 9.24±7.73  11.7±7.03 | 46.5 | 0.372^#^ |
| Extension of the lesion | Single  Multiple | 12(40)  18(60) | 7.38±5.97  14.0±6.61 | 48.5 | 0.012^#^* |
| Type of OPMDs | OSF  LKP | 15(50)  15(50) | 16.77±5.33  5.94±3.53 | 7 | <0.001^#^* |
| Epithelial dysplasia | Absent  Present | 18(60)  12(40) | 13.36±6.89  8.35±6.50 | 62.5 | 0.054^#^ |
| Parameters | **Category** | **n%** | **ST2 (Mean ± SD)** | **U statistic** | ***p*-value** |
| Age | ≤45  >45 | 8(27)  22(73) | 892.83±264.27  758.90±215.47 | 57 | 0.146^#^ |
| Sex | Male  Female | 24(80)  6(20) | 792.91±240.70  801.43±217.36 | 70 | 0.917^#^ |
| Habits | Absent  Present | 5(17)  25(83) | 738.10±209.21  805.92±239.34 | 51 | 0.522^#^ |
| Extension of the lesion | Single  Multiple | 12(40)  18(60) | 760.87±180.67  817.115±264.27 | 91 | 0.472^#^ |
| Type of OPMDs | OSF  LKP | 15(50)  15(50) | 933.56±184.30  655.67±191.68 | 33 | 0.001^#^* |
| Epithelial dysplasia | Absent  Present | 18(60)  12(40) | 847.65±254.23  715.06±176.78 | 70 | 0.108^#^ |
| Parameters | **Category** | **n%** | **IL-10 (Mean ± SD)** | **U statistic** | ***p*-value** |
| Age | ≤45  >45 | 8(27)  22(73) | 13.94±4.33  12.29±4.95 | 70 | 0.396^#^ |
| Sex | Male  Female | 24(80)  6(20) | 12.80±4.96  12.45±4.39 | 67.5 | 0.815^#^ |
| Habits | Absent  Present | 5(17)  25(83) | 8.90±4.01  13.5±4.61 | 22.5 | 0.025^#^* |
| Extension of the lesion | Single  Multiple | 12(40)  18(60) | 10.08±4.40  14.50±4.26 | 48.5 | 0.011^#^* |
| Type of OPMDs | OSF  LKP | 15(50)  15(50) | 17.11±1.85  8.36±1.71 | <0.1 | <0.001^#^* |
| Epithelial dysplasia | Absent  Present | 18(60)  12(40) | 13.95±4.77  10.91±4.35 | 73.5 | 0.142^#^ |
| Parameters | **Category** | **n%** | **IFN-γ (Mean ± SD)** | **U statistic** | ***p*-value** |
| Age | ≤45  >45 | 8(27)  22(73) | 27.24±13.12  28.29±10.75 | 87.5 | 0.981^#^ |
| Sex | Male  Female | 24(80)  6(20) | 28.01±10.87  27.47±13.59 | 66.5 | 0.774^#^ |
| Habits | Absent  Present | 5(17)  25(83) | 31.87±8.83  27.2±11.62 | 50 | 0.484^#^ |
| Extension of the lesion | Single  Multiple | 12(40)  18(60) | 31.32±8.97  25.8±12.21 | 79.5 | 0.225^#^ |
| Type of OPMDs | OSF  LKP | 15(50)  15(50) | 17.67±1.92  38.35±5.21 | <0.1 | <0.001^#^**^*^** |
| Epithelial dysplasia | Absent  Present | 18(60)  12(40) | 26.37±11.48  30.46±10.79 | 89.5 | 0.431^#^ |
| ^#^ MWU test; * Significance | | | |  |  |

| **Table 5. Raw data - Serum cytokine IL-33, ST2, IL-10 and IFN-Ƴ levels in Health, OPMDs and OSCC groups** | | | | | | | | | | | | | | |
| --- | --- | --- | --- | --- | --- | --- | --- | --- | --- | --- | --- | --- | --- | --- |
| **G-1** | **IL-33** | **ST2** | **IL-10** | **INF-Ƴ** | **G2** | **IL-33** | **ST2** | **IL-10** | **IFN-Ƴ** | **G3** | **IL-33** | **ST2** | **IL-10** | **IFN-Ƴ** |
| 1 | 2.916 | 481.928 | 4.7352 | 25.0873 | 1 | 10.5 | 698.3571 | 18.1176 | 18.5793 | 1 | 29.333 | 1099.964 | 24.3529 | 13.8253 |
| 2 | 4.33 | 451.928 | 4.3825 | 24.7777 | 2 | 4.0833 | 694.8571 | 7.2058 | 34.4841 | 2 | 32.833 | 1389.036 | 28.0882 | 14.492 |
| 3 | - | 277 | 4.8235 | 21.1428 | 3 | - | 660.9643 | 6.8529 | 32.5238 | 3 | 37.416 | 1736.107 | 46.9117 | 9.4523 |
| 4 | - | 527.607 | 3.8235 | 18.8095 | 4 | 9.1666 | 601.0357 | 17.7941 | 17.9206 | 4 | 21.833 | 1156.929 | 18.7352 | 15.2539 |
| 5 | 5.75 | 277.571 | 4.5882 | 25.3333 | 5 | 21 | 1129.25 | 18.2941 | 18.3888 | 5 | 36.166 | 1233.679 | 18.5882 | 15.1349 |
| 6 | 3.5 | 474.571 | 6.2058 | 30.6746 | 6 | 9.583 | 966.4643 | 7.5882 | 34.2698 | 6 | 35.58 | 1735.75 | 52.7647 | 10.246 |
| 7 | - | 663 | 4.7647 | 23.238 | 7 | 10.08 | 913.5357 | 7.647 | 33.992 | 7 | 46.083 | 1861.929 | 44.3529 | 10.6031 |
| 8 | - | 702.714 | 3.6176 | 17.5793 | 8 | 6.75 | 379.5714 | 10.8823 | 45.1031 | 8 | 23.5 | 1125.357 | 18.7941 | 10.9555 |
| 9 | 6.833 | 246.857 | 5.5294 | 28.8253 | 9 | 9.5 | 870.3929 | 10.4705 | 46.9047 | 9 | 20.833 | 1131.75 | 19.3529 | 10.9523 |
| 10 | - | 567.25 | 4.2058 | 19.7222 | 10 | 3.25 | 817.5714 | 7.6764 | 34.0793 | 10 | 21.333 | 1264.857 | 21.5294 | 15.0555 |
| 11 | 2.833 | 561.285 | 3.5882 | 17.2936 | 11 | 13.666 | 976.3929 | 19.3823 | 19.4523 | 11 | 32.833 | 1540.321 | 19.2352 | 16.3333 |
| 12 | - | 200.142 | 4.0882 | 21.1031 | 12 | 16 | 1032.107 | 14.7352 | 15.373 | 12 | 34.583 | 1593.75 | 50.7941 | 9.8492 |
| 13 | - | 496.928 | 2.3529 | 10.0634 | 13 | 28.5 | 1102.143 | 14.4705 | 14.7936 | 13 | 24.666 | 1360.321 | 21.7941 | 15.1507 |
| 14 | 5 | 494.321 | 6.1176 | 30.7142 | 14 | 6.666 | 465.6786 | 9.4117 | 43.0714 | 14 | 22.166 | 1537.429 | 19.8529 | 18.2936 |
| 15 | 8.333 | 545.678 | 4.0294 | 21.4841 | 15 | 22 | 946.0714 | 16.0588 | 16.3492 | 15 | 46.666 | 1676.857 | 52.8529 | 10.5158 |
| 16 | - | 461.535 | 4.3529 | 24.5238 | 16 | 18.75 | 1162.964 | 17.4411 | 18.6666 | 16 | 38.083 | 1735.035 | 45.6764 | 9.4126 |
| 17 | 5.0833 | 245.71 | 5.764 | 32.1825 | 17 | 18.1666 | 827.7857 | 16.8529 | 18.2698 | 17 | 21.166 | 1447.393 | 27.0882 | 16.1666 |
| 18 | 9.5 | 625.785 | 4.7352 | 27.1746 | 18 | 22.666 | 692.9286 | 15.7647 | 15.7857 | 18 | 46.25 | 1571.179 | 49.7941 | 10.238 |
| 19 | 3.16 | 241.5 | 6.4117 | 34.2936 | 19 | 11.916 | 1122.964 | 16.2941 | 16.4126 | 19 | 36.333 | 1544.036 | 45.4705 | 10.2857 |
| 20 | - | 452.678 | 5.1764 | 30.2777 | 20 | 11.25 | 887.8571 | 12.0294 | 46.7301 | 20 | 45.666 | 1551.179 | 43.0294 | 9.7857 |
| 21 | - | 212.142 | 7.2647 | 35.0476 | 21 | 18.25 | 1096.357 | 17.2647 | 18.8492 | 21 | 21.666 | 1392.821 | 22.7058 | 11.738 |
| 22 | 10.666 | 670.821 | 5.6176 | 33.619 | 22 | 2.25 | 558.1786 | 7.0588 | 38.4523 | 22 | 24.666 | 1214.607 | 19.2058 | 13.5079 |
| 23 | 9.75 | 643.892 | 4.1176 | 22.5793 | 23 | 16 | 806.6071 | 15.8235 | 16.6746 | 23 | 19.75 | 1155.214 | 22.9705 | 11.8095 |
| 24 | 7.583 | 443.607 | 6.0882 | 35.5555 | 24 | 14.25 | 783.25 | 21.7647 | 22.4285 | 24 | 22.75 | 1175.786 | 21.9411 | 16.6984 |
| 25 | 11.25 | 476.642 | 4.647 | 22.9206 | 25 | - | 582.2143 | 6.1764 | 34.1349 | 25 | 23.916 | 1213.964 | 20.3235 | 18.0634 |
| 26 | 7.58 | 479.5 | 5.647 | 30.8095 | 26 | 4.75 | 544.8929 | 7.9411 | 33.746 | 26 | 34.25 | 1295.983 | 20.1764 | 15.2063 |
| 27 | 10.5 | 617.607 | 4.4411 | 21.5634 | 27 | 10.833 | 1025.321 | 16.6176 | 17.0634 | 27 | 21.416 | 1462.357 | 22.7647 | 15.119 |
| 28 | 6.166 | 580.571 | 5.529 | 31.8412 | 28 | 8.333 | 524.9286 | 6.6176 | 35.8095 | 28 | 40.166 | 1678.607 | 43.9411 | 11.8968 |
| 29 | 9.25 | 568.25 | 5.4117 | 29.5476 | 29 | 6.41 | 547.2143 | 9.1764 | 42.9528 | 29 | 32.25 | 1727.107 | 45.2352 | 10.1269 |
| 30 | 5.833 | 474.392 | 5.647 | 31.4603 | 30 | 6.33 | 420.75 | 8.7058 | 39.1111 | 30 | 40.75 | 1486.786 | 47.7647 | 10.5317 |
